# Diminished seroconversion following a single SARS-COV-2 vaccine in ocrelizumab-treated relapsing-remitting multiple sclerosis patients

**DOI:** 10.1177/13524585211046786

**Published:** 2021-10-01

**Authors:** Zoya G Georgieva, Rainer Dӧffinger, Dinakantha Kumararatne, Alasdair J Coles, Claire McCarthy

**Affiliations:** Department of Clinical Neurosciences, University of Cambridge, Hills Road, Cambridge CB2 0AH, UK; Department of Clinical Neurosciences, University of Cambridge, Cambridge, UK; Department for Clinical Biochemistry and Immunology, Cambridge University Hospital, Cambridge, UK; Department for Clinical Biochemistry and Immunology, Cambridge University Hospital, Cambridge, UK; Department of Clinical Neurosciences, University of Cambridge, Cambridge, UK; Department of Clinical Neurosciences, Cambridge University Hospital, Cambridge, UK

**Keywords:** Ocrelizumab, SARS-COV-2, COVID-19, vaccination, antibodies

## Abstract

**Background:**

Despite impressive efficacy in immunocompetent individuals, the immunogenicity of a single dose of COVID-19 vaccine in B-cell-deplete patients remains unknown.

**Objectives:**

We aimed to quantify real-world vaccine immunogenicity in ocrelizumab recipients.

**Methods:**

We measured post-vaccination SARS-COV-2 immunoglobulin G (IgG) in ocrelizumab recipients using a highly sensitive Luminex assay.

**Results:**

44.1% of patients had detectable SARS-COV-2-IgG 21+ days after one vaccine dose, regardless of vaccine type (AZD1222 vs BNT162b2, odds ratio (OR) = 0.62, 95% confidence interval (CI) = 0.157–2.32, *p* = 0.72). B-cell count strongly predicted seroconversion (β1 = 12.38, 95% CI = 4.59–20.16, *p* = 0.0029), but undetectable B-cells did not preclude it. The second vaccine seroconverted 53% of the patients who had not already responded to dose 1.

**Conclusion:**

Humoral response after one COVID-19 vaccine dose is lower than expected in CD20-deplete patients.

## Introduction

Patients taking immunosuppressive medication have been prioritised for SARS-COV-2 vaccination.^1^ The efficacy of several SARS-COV-2 vaccines has been demonstrated in immunocompetent individuals, emphasising humoral immunity outcomes.^2,3^ However, the efficacy of a single vaccine dose in B-cell-depleted patients remains unknown. To establish whether they mount a humoral immune response, we measured SARS-COV-2 IgG seroconversion post-vaccination.

## Methods

We audited the outcomes of SARS-COV-2 vaccination in individuals treated with ocrelizumab, as per section 4.4 of the ocrelizumab SmPC^4^ and UK government ‘Green Book’ guidance.^5^

Between January and April 2021, all patients receiving ocrelizumab for relapsing-remitting MS at a single centre were invited for blood tests as part of medical care, including extended lymphocyte phenotyping, immunoglobulins and SARS-COV2-IgG. Patients received either the BNT162b2 or AZD1222 SARS-COV2 vaccine via the UK National Health Service as standard medical care for people with MS. Patients were asked to give blood at baseline (within ±3 days of vaccine dose 1) and again 21+ days after the first vaccine. Individuals who were seronegative after one vaccine dose were offered a repeat SARS-COV-2 IgG test after the second dose.

The Luminex-based SARS-COV2-IgG assay has been previously described.^6^ Briefly, carboxylated beads covalently coupled to SARS-COV-2 peptides capture human IgG against SARS-COV-2 receptor-binding domain (R), trimeric spike protein (S) and nucleocapsid protein (N). Beads were incubated with patient sera (1/100 dilution) for 1 hour in 96-well filter plates (MultiScreenHTS; Millipore) at room temperature in the dark on a horizontal shaker. Beads were washed three times with 10 mM PBS/0.05% Tween 20, incubated for 30 minutes with a Phycoerythrin (PE)-labelled anti-human IgG-Fc antibody (Leinco/Biotrend), washed again and re-suspended in 100 μL PBS/Tween. They were analysed on a Luminex analyser (Luminex/R&D Systems) using Exponent Software V31. Positivity was defined by receiver operating characteristic (ROC) analysis as S-antibody levels exceeding 1896 median fluorescence intensity (MFI); the threshold for R-antibody was 456 MFI, and for N-antibody was 6104 MFI.

This assay has been validated on patients with polymerase chain reaction (PCR)-confirmed SARS-COV-2 infection, in whom it is 84% sensitive and 100% specific. Cross-reactivity with healthy control pre-pandemic sera is very rare (due to similar N-protein in other *Coronaviridae*). Immunocompetent vaccinated patients typically test positive for anti-S and/or receptor-binding domain (RBD), but not anti-N antibodies. Previous infection results in dual-positive anti-S and N antibodies.

Lymphocyte counts were measured by standard clinical flow cytometry on whole blood, contemporaneous with pre-vaccine SARS-COV-2 IgG testing. Data were analyzed in the GraphPad Prism v8.3.0 using the two-tailed Mann–Whitney or Fisher’s exact test. Results were considered significant at alpha = 0.05, equivalent to a Bonferroni-adjusted *p* < 0.0125; we show unadjusted *p*-values. Exploratory analysis was performed using the least-squares multiple logistic regression. Candidate models were assessed for goodness-of-fit using the Akaike Information Criterion (AICc). Data are presented as mean value ± standard error of the mean (SEM) unless specified.

## Results

Patient characteristics and numbers are summarised in Table 1.

**Table 1.** Patient characteristics and inclusion in analyses.

Total patient population, *N* = 110	
Reason for attrition	
Unable/unwilling to attend	39 (80%)
Declined vaccination now	5 (10%)
Ocrelizumab <4 weeks ago	2 (4%)
Provided blood only after second dose	3 (6%)
Patients with SARS-COV2-IgG result after first vaccine, *N* = 61	
Age^a^	40.0 years (17–59)
Sex (F)	45 (71%)
Commenced ocrelizumab^a^	16.25 months (2.25–55)
Time since last ocrelizumab^a^	5 months (1.25–7)
Patients with matched baseline/post first vaccine SARS-COV2-IgG result, *N* = 38	
Age^a^	37 years (17–59)
Sex (F)	27 (71%)
Time since last ocrelizumab^a^	5 months (1.25–7)
Seronegative before first vaccine dose	34
Vaccine type^b^	AZD122 = 17 BNT162b2 = 15 Unknown = 2
Seropositive before first vaccine dose (past infection)	4
Patients with available SARS-COV2-IgG result after vaccine dose 2 (who had failed to respond to the first vaccine) *N* = 15	

a Data are expressed as median (range).

b AZD1222 Oxford–AstraZeneca vaccine; BNT162b2 Pfizer–BioNTech vaccine.

Matched pre- and post-single-vaccination SARS-COV2-IgG results were available for 38/61 patients, of whom 34 were seronegative before vaccination. Baseline blood was donated just before vaccination (median = 1 day and interquartile range (IQR) = 2 days). Median time from first vaccination till SARS-COV2-IgG testing was 28 days (IQR = 4 days and range = 20–70 days).

**Figure 1.**
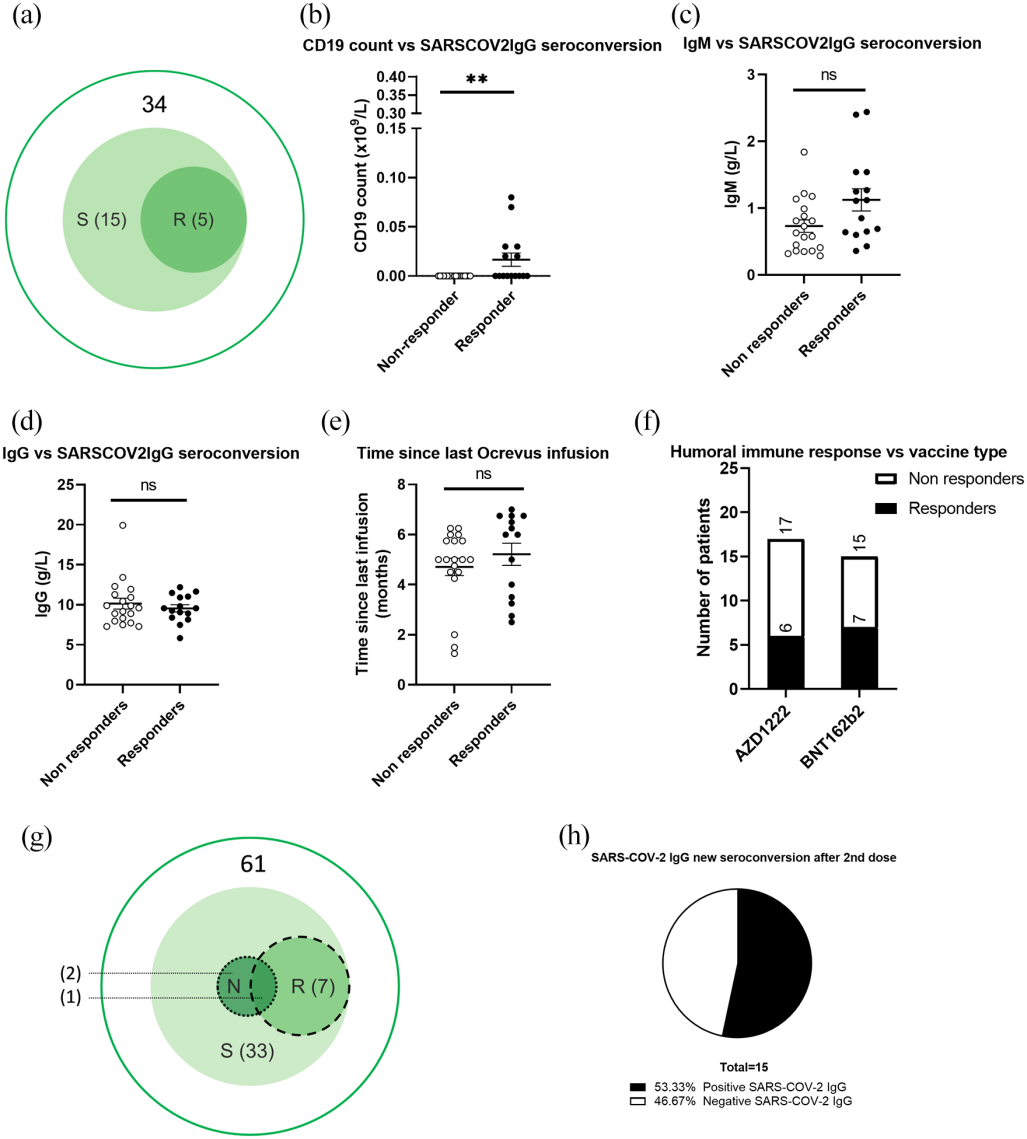
(a) Detectable SARS-COV-2 IgG (by target epitope) after 1 vaccine dose in patients who were known seronegative before vaccination (*N* = 34). (b)–(e) Univariate analyses of CD19 count, IgM, IgG and interval between vaccine dose 1 and ocrelizumab infusion (subgroup with matched pre-/post-first vaccine bloods, *N* = 34). (f) Effect of vaccine type (after 1st dose). (g) Detectable SARS-COV-2 IgG after 1 vaccine dose in all patients (irrespective of pre-vaccination result). (h) Seroconversion after second vaccine dose in patients who had not already responded to the first dose (*N* = 15). S: spike protein; R: receptor-binding domain; N: nucleocapsid protein; AZD1222: Oxford–AstraZeneca vaccine; BNT162b2: Pfizer–BioNTech vaccine.

44.1% of patients (15/34) developed SARS-COV2-IgG 21+ days after the first vaccine (Figure 1(a)). Of the four patients seropositive before vaccination (3 = S+/N+; 1 = S+): two developed new RBD antibodies, one remained S-positive and one patient (weakly S-positive at baseline) tested negative after vaccination (not shown).

Responders were significantly more likely to have detectable CD19+ B-lymphocytes (0.00 × 10^9^/L vs 0.0167 × 10^9^/L, respectively, *p* = 0.0037, two-tailed Mann–Whitney test, Figure 1(b)). Univariate analysis showed no significant difference between non-responders and responders in total IgG (10.15 ± 0.67 vs 9.56 ± 0.44, *p* = 0.92), IgM (0.73 ±0.09 vs 1.12 ±0.16, *p* = 0.039) or time since the latest ocrelizumab infusion (4.7 ± 0.34 vs 5.2 ± 0.44 months, *p* = 0.18) (Figure 1(c)–(e)). Vaccine type did not influence the odds of producing antibodies after one dose (odds ratio (OR) = 0.62, 95% confidence interval (CI) = 0.157–2.32, *p* = 0.72) (Figure 1(f)).

Considering all patients with available SARS-COV2-IgG 21+ days after 1 vaccine dose, 54% (33/61) had detectable SARS-COV-2-IgG. 23/61 were S+ only and 7/61 S/RBD+. 3/61 were additionally N-positive (indicating past infection; Figure 1(g)). Post-second-vaccination SARS-COV-2 IgG results were available for 15 patients who had failed to respond to the first dose. 8/15 (53%) developed new antibodies after the second vaccine dose (Figure 1(h)).

In exploratory logit analysis, CD19 count remained a strong predictor of successful seroconversion after 1 vaccine dose (β1 = 12.38, 95% CI = 4.59–20.16, *p* = 0.0029). There was also a weak signal for IgM titre (β2 = 0.33, 95% CI = 0.064–0.6, *p* = 0.0168). Sequential pairwise comparisons of different logit models showed this to be the best one utilising our available parameters (AICc = −51.4) but goodness-of-fit was overall poor (adjusted *R*^2^ = 0.359, degree of freedom (df) = 30).

## Discussion

We aimed to determine if patients treated with the B-cell-depleting drug ocrelizumab produce a detectable humoral immune response following SARS-COV-2 vaccination. We observed an attenuated early humoral immune response in this population following one vaccine dose.

44.1% of ocrelizumab-treated patients developed SARS-COV2-IgG 21+ days after dose 1 of the vaccine. By contrast, most phase 1/2 trial participants receiving one dose of BNT162b2 vaccine had detectable anti-S IgG at day 21 (11/11 aged = 18–55 years; 11/12 aged = 65–85 years).^7^ 91%–100% of participants developed neutralising antibodies 28 days after one dose of AZD1222^8^ and 79% of patients developed neutralising antibodies after one BNT162b dose.^9^ Here, we measured the presence of S-, N- and/or RBD-antibodies (not neutralisation). Even accounting for false-negatives (16%), we observed a diminished rate of SARS-COV-2-IgG seroconversion after 1 vaccine dose. However, the second vaccine generated antibodies in 53% of the patients who had originally not developed detectable antibodies after one dose.

Our findings are consistent with previous observations of diminished immunity to tetanus toxoid and pneumococcal antigen in ocrelizumab recipients.^10^ A controlled study in 23 ocrelizumab recipients showed humoral immunity in only 22% patients after two SARS-COV-2 vaccine doses.^11^

In our cohort, detectable B-cells strongly predicted SARS-COV-2 seroconversion, but the absence of circulating B-cells did not preclude it. Furthermore, T-cell-mediated immunity is likely to be an important unmeasured protective factor. Vaccination with AZD1222 produces a peak Th-1 skewed response 14 days after one dose,^8^ while RBD-specific CD4+ Th1-cells are detectable 7 days after the second dose of BNT162b1.^12^ This should not differ substantially in B-cell deplete individuals.

This study adds to mounting evidence for diminished early humoral immunity in CD20-deplete patients. The contribution of cellular immunity and booster vaccination remains to be fully assessed.

## References

[1] JCVI. Guidance on shielding and protecting CEV persons from COVID-19, https://www.gov.uk/government/publications/guidance-on-shielding-and-protecting-extremely-vulnerable-persons-from-covid-19/guidance-on-shielding-and-protecting-extremely-vulnerable-persons-from-covid-19#cev (2021, accessed 6 April 2021).

[2] Ramasamy MN Minassian AM Ewer KJ , et al. Safety and immunogenicity of ChAdOx1 nCoV-19 vaccine administered in a prime-boost regimen in young and old adults (COV002): A single-blind, randomised, controlled, phase 2/3 trial. Lancet 2020; 396: 1979–1993. 33220855 10.1016/S0140-6736(20)32466-1 PMC7674972

[3] Polack FP Thomas SJ Kitchin N , et al. Safety and efficacy of the BNT162b2 mRNA COVID-19 vaccine. N Engl J Med 2020; 383: 2603–2615. 33301246 10.1056/NEJMoa2034577 PMC7745181

[4] OCREVUS 300 mg concentrate for solution for infusion: Summary of Product Characteristics (SmPC) – (emc), https://www.medicines.org.uk/emc/product/8898/smpc#gref (accessed 5 July 2021).

[5] Public Health England. Greenbook chapter 14A COVID-19, https://assets.publishing.service.gov.uk/government/uploads/system/uploads/attachment_data/file/998309/Greenbook_chapter_14a_1July2021.pdf (2021, accessed 5 July 2021).

[6] Xiong X Qu K Ciazynska KA , et al. A thermostable, closed SARS-CoV-2 spike protein trimer. Nat Struct Mol Biol 2020; 27(10): 934–941. 32737467 10.1038/s41594-020-0478-5 PMC7116388

[7] Walsh EE Frenck RW Falsey AR , et al. Safety and immunogenicity of two RNA-based COVID-19 vaccine candidates. N Engl J Med 2020; 383: 2439–2450. 33053279 10.1056/NEJMoa2027906 PMC7583697

[8] Folegatti PM Ewer KJ Aley PK , et al. Safety and immunogenicity of the ChAdOx1 nCoV-19 vaccine against SARS-CoV-2: A preliminary report of a phase 1/2, single-blind, randomised controlled trial. Lancet 2020; 396: 467–478. 32702298 10.1016/S0140-6736(20)31604-4 PMC7445431

[9] Collier D. Age-related heterogeneity in neutralising antibody responses to SARS-CoV-2 following BNT162b2 vaccination. SSRN, 10.2139/ssrn.3782450 (accessed 21 April 2021).

[10] Bar-Or A Calkwood JC Chognot C , et al. Effect of ocrelizumab on vaccine responses in patients with multiple sclerosis: The VELOCE study. Neurology 2020; 95: e1999–e2008. 32727835 10.1212/WNL.0000000000010380 PMC7843152

[11] Achiron A Mandel M Dreyer-Alster S , et al. Humoral immune response to COVID-19 mRNA vaccine in patients with multiple sclerosis treated with high-efficacy disease-modifying therapies. Ther Adv Neurol Disord 2021; 14: 1012836. 10.1177/17562864211012835 PMC8072850 34035836

[12] Sahin U Muik A Derhovanessian E , et al. COVID-19 vaccine BNT162b1 elicits human antibody and TH1 T cell responses. Nature 2020; 586: 594–599. 32998157 10.1038/s41586-020-2814-7

